# Albumin-induced apoptosis of tubular cells is modulated by BASP1

**DOI:** 10.1038/cddis.2015.1

**Published:** 2015-02-12

**Authors:** M D Sanchez-Niño, B Fernandez-Fernandez, M V Perez-Gomez, J Poveda, A B Sanz, P Cannata-Ortiz, M Ruiz-Ortega, J Egido, R Selgas, A Ortiz

**Affiliations:** 1 Instituto de Investigacion Sanitaria IDIPAZ, Madrid, Spain; 2 REDINREN, Madrid, Spain; 3 IIS-Fundación Jiménez Díaz-Universidad Autónoma de Madrid and Fundación Renal Iñigo Alvarez de Toledo-IRSIN, Madrid, Spain

## Abstract

Albuminuria promotes tubular injury and cell death, and is associated with faster progression of chronic kidney disease (CKD) to end-stage renal disease. However, the molecular mechanisms regulating tubular cell death in response to albuminuria are not fully understood. Brain abundant signal protein 1 (BASP1) was recently shown to mediate glucose-induced apoptosis in tubular cells. We have studied the role of BASP1 in albumin-induced tubular cell death. BASP1 expression was studied in experimental puromycin aminonucleoside-induced nephrotic syndrome in rats and in human nephrotic syndrome. The role of BASP1 in albumin-induced apoptosis was studied in cultured human HK2 proximal tubular epithelial cells. Puromycin aminonucleoside induced proteinuria and increased total kidney BASP1 mRNA and protein expression. Immunohistochemistry localized the increased BASP1 to tubular cells. BASP1 expression colocalized with deoxynucleotidyl-transferase-mediated dUTP nick-end labeling staining for apoptotic cells. Increased tubular BASP1 expression was observed in human proteinuric nephropathy by immunohistochemistry, providing evidence for potential clinical relevance. In cultured tubular cells, albumin induced apoptosis and increased BASP1 mRNA and protein expression at 6–48 h. Confocal microscopy localized the increased BASP1 expression in albumin-treated cells mainly to the perinuclear area. A peripheral location near the cell membrane was more conspicuous in albumin-treated apoptotic cells, where it colocalized with actin. Inhibition of BASP1 expression by a BASP1 siRNA protected from albumin-induced apoptosis. In conclusion, albumin-induced apoptosis in tubular cells is BASP1-dependent. This information may be used to design novel therapeutic approaches to slow CKD progression based on protection of tubular cells from the adverse consequences of albuminuria.

Chronic kidney disease (CKD) is associated with adverse patient outcomes, either as a consequence of increased cardiovascular mortality or of progression to end-stage renal disease.^1^ Proteinuria in CKD is mainly composed of albumin. Pathological albuminuria is now used for CKD risk stratification, because it is associated both with increased cardiovascular mortality and accelerated progression of CKD.^2, 3^ In fact, the only known nephroprotective drugs that slow CKD progression are anti-proteinuric drugs. However, current anti-proteinuric strategies may have adverse effects that limit their protein-lowering and nephroprotective potential.^4^ Furthermore, residual albuminuria in patients already treated with anti-proteinuric drugs is still associated with worse outcomes. A better understanding of the molecular mechanisms linking albuminuria to CKD progression may offer the chance to develop novel nephroprotective strategies. In this regard, albuminuria and tubulointerstitial injury are among the key outcome indicators in glomerular diseases and evidence suggests a role of albuminuria in promoting tubular injury and subsequent interstitial inflammation and fibrosis.^3, 5, 6, 7^ Cell culture and animal models have identified several deleterious effects of albuminuria or albumin on kidney tubular epithelial cells.^8, 9, 10, 11, 12, 13, 14^ Animal models of albuminuria resulting from albumin overload are associated with tubular cell death and tubulointerstitial inflammation and eventual fibrosis.^15, 16^ Numerous cell culture studies have described a pro-apoptotic and pro-inflammatory role of albumin on tubular epithelial cells.^10, 11, 12, 13, 14^ PKC-delta activation, oxidative stress, endoplasmic reticulum stress and caspase-8 activation have been described as potential mechanisms mediating albumin-induced apoptosis. However, the molecular mechanisms regulating tubular cell death in response to albuminuria are not fully understood.

Apoptosis is an active response to an altered microenvironment characterized by the activation of specific intracellular lethal pathways.^17^ The presence of injurious factors and/or the lack of survival factors may activate the apoptotic molecular machinery. The involvement of specific molecules that are activated or suppressed allows the design of therapeutic strategies that modulate the expression or activity of apoptosis regulatory factors.^18^ Brain-abundant, membrane-attached signal protein 1 (BASP1) was recently characterized as an intracellular proapoptotic factor that was required for high glucose-induced apoptosis in kidney proximal tubular cells.^19^ BASP1 is a 23-kDa myristoylated protein originally isolated from brain extracts^20, 21^ that shares 70% homology in human and rat.^22^ A transcriptomics approach disclosed that BASP1 expression was increased in human diabetic nephropathy tubulointerstitium.^19^ Immunohistochemistry localized the increased BASP1 expression to tubular cells both in humans and in experimental diabetes. Interestingly, not all tubules were BASP1-positive: there were BASP1-positive and BASP1-negative tubules within the same diabetic nephropathy biopsy. Cell culture studies identified a high glucose concentration as an inducer of BASP1 expression and BASP1-dependent apoptosis. However, this cell culture observation does not explain the patchy distribution of BASP1-expressing tubules in human diabetic kidney, as all tubules would have been exposed to the same high glucose concentrations. As albumin induces apoptosis in cultured tubular cells,^10, 11, 12^ we hypothesized that albumin could be an inducer of BASP1 expression in tubular cells. This hypothesis might explain the observation of BASP1-positive and -negative tubules in human diabetic nephropathy and experimental diabetic and hypertensive nephropathies.^19^ Tubules belonging to a nephron where podocyte injury has resulted in glomeruloesclerosis and more severe albuminuria would be expected to express more BASP1 than tubules from nephrons with more preserved functional podocytes. To explore this hypothesis, we used cell culture and non-diabetic albuminuria models. We now report that albumin induces Basp1 expression in cultured proximal tubular cells and in tubular cells *in vivo* and that targeting Basp1 protects tubular cells from albumin-induced apoptosis. This information may be used to protect the tubulointerstitium from structural damage in proteinuric kidney diseases with residual albuminuria despite anti-proteinuric therapy.

## Results

### PAN induces nephrotic syndrome and BASP1 expression in the rat kidney

**Figure 1:**
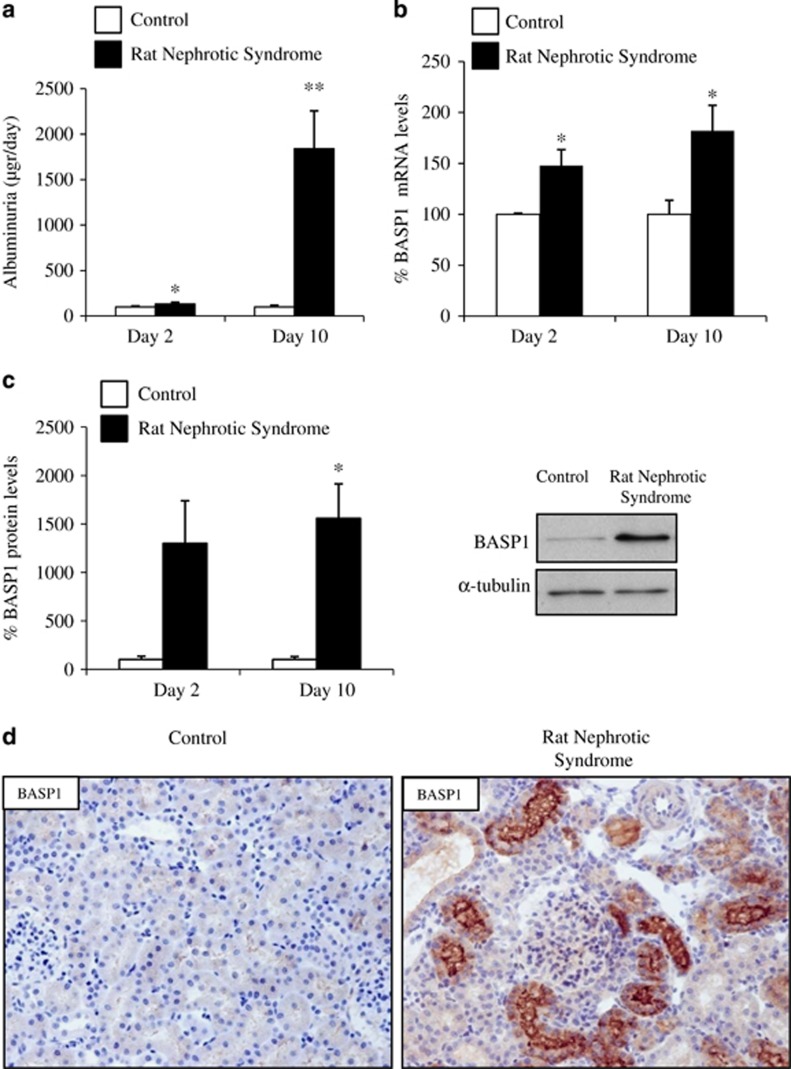
Tubular cell BASP1 expression is increased in rat nephrotic syndrome induced by PAN. (**a**) Systemic PAN administration resulted in nephrotic syndrome with severe albuminuria (shown), which was fully established at day 10. At that time-point, other features of nephrotic syndrome were present such as hypercholesterolemia, hypoalbuminemia and ascites (not shown). *P<0.02 *versus* control, **P<0.005 *versus* control. (**b**) Systemic PAN administration increased whole-kidney BASP1 mRNA expression at day 2 and 10 as assessed by RT-qPCR. *P<0.05 *versus* control. (**c**) Systemic PAN administration increased whole-kidney BASP1 protein expression at day 2 and 10. Quantification and representative image of western blot of whole-kidney extracts. *P<0.001 *versus* control. (**d**) Immunohistochemistry with anti-BASP1 antibody localized BASP1 to tubular cells at day 10 following PAN administration. Original magnification × 20

**Figure 2:**
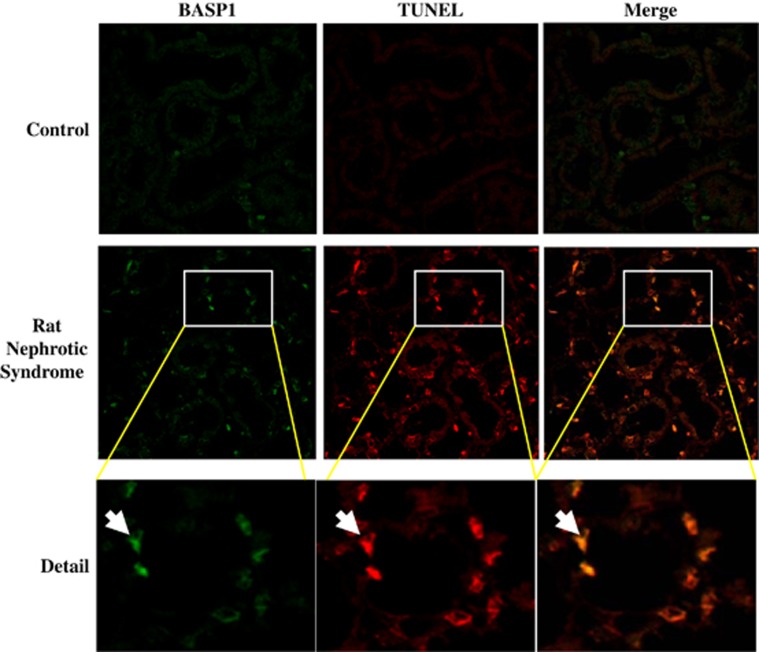
Colocalization of tubular cell BASP1 with TUNEL-positive cells in rat nephrotic syndrome induced by PAN. BASP1 immunofluorescence positive cells (green) colocalized with cells stained with TUNEL (red) for fragmented DNA characteristic of apoptosis (arrows) at day 10. Original magnification × 20. Detail × 60

Systemic puromycin aminonucleoside (PAN) administration causes podocyte injury in rats leading to an increased urinary protein excretion at day 2 and full-blown nephrotic syndrome characterized by severe albuminuria (Figure 1a), hypoalbuminemia, hypercholesterolemia and ascites at day 10. Increased whole-kidney BASP1 mRNA (Figure 1b) and protein (Figure 1c) expression was noted 2 and 10 days post PAN injection, coinciding onset and progression to severe albuminuria. BASP1 protein was localized diffusely to tubular epithelial cells by immunohistochemistry (Figure 1d). Furthermore, tubular cell BASP1 expression colocalized with deoxynucleotidyl-transferase-mediated dUTP nick-end labeling-positive cells, indicating expression of BASP1 by tubular cells undergoing apoptosis (Figure 2).

### Increased BASP1 expression in human proteinuric nephropathy

**Figure 3:**
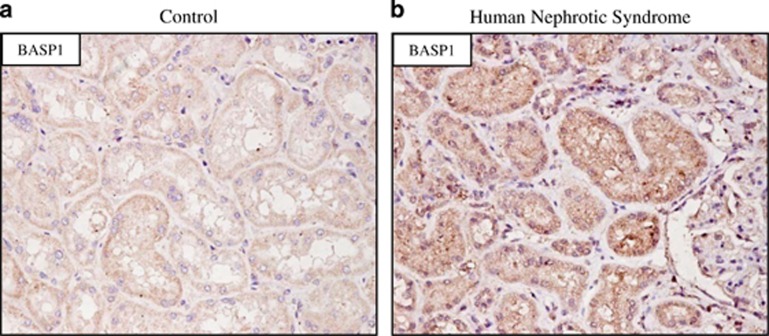
Increased BASP1 expression in tubular cells in human nephrotic syndrome. BASP1 immunohistochemistry. (**a**) Minimal tubular BASP1 staining was observed in control kidney tubules. (**b**) BASP1-positive tubular cells were observed in human nephrotic syndrome. Original magnification × 20

In human proteinuric nephropathy, immunohistochemistry identified BASP1-positive tubular cells (Figure 3). Minimal BASP1 staining was observed in tubules from control kidney samples.

### Tubular cell death induced by albumin has features of apoptosis

**Figure 4:**
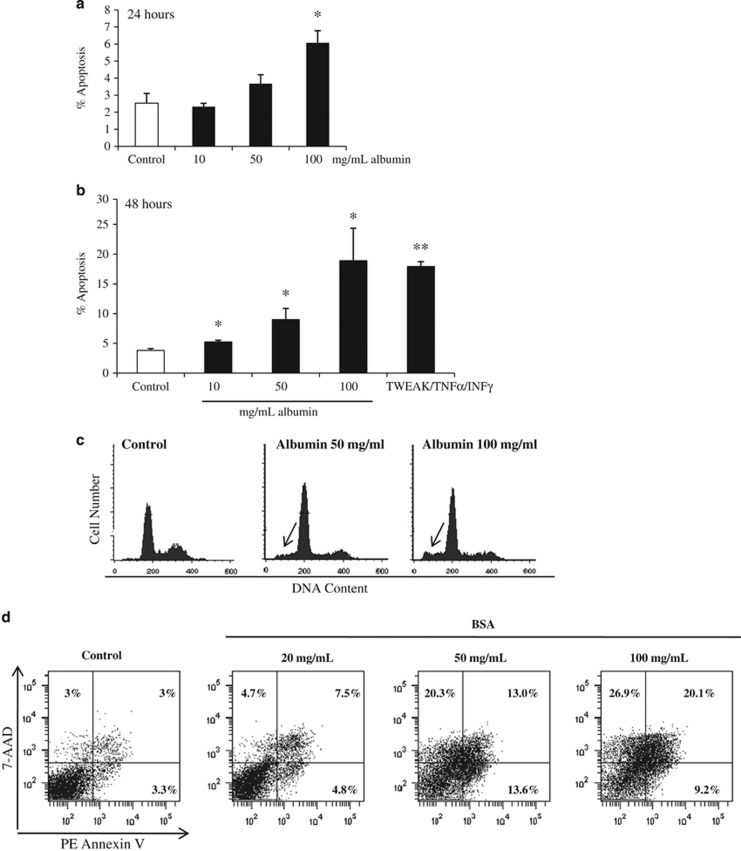
Albumin induces apoptosis in human proximal tubular epithelial cells. Albumin-induced apoptosis in tubular cells was time- and dose-dependent. (**a**) Dose–response at 24 h. *P<0.05 *versus* control. (**b**) Dose–response at 48 h. Cell death was assessed by flow cytometry of DNA content (hypodiploid cells suggestive of apoptosis). Note the difference in scale between 24 h and 48 h data. Cells exposed to proinflammatory cytokines (100 ng/ml TWEAK, 30 ng/ml TNF*α* and INF-*γ* 30 U/ml –TTI-) for 48 h were used as a positive control. *P<0.05 *versus* control, **P<0.02 *versus* control. (**c**) Representative images of DNA content flow cytometry results at 48 h. Note an increase in hypodiploid apoptotic cells in albumin-treated cells (arrows). Mean±S.D. of three independent experiments. (**d**) Cell death was also assessed by flow cytometry following staining with annexin V and 7-AAD after culture in the presence of albumin for 48 h. Representative experiment

Under physiological conditions, proximal tubular cells reabsorb the minimal amounts of albumin that may get through the glomerular filtration barrier. The combination of albumin retention in plasma by the glomerular filtration barrier and proximal tubular cell reabsorption results in minimal (<30 mg per day in humans) physiological albuminuria. However, glomerular injury may be associated with filtration of massive amounts of albumin into the tubular lumen. Such high amounts of albumin promote proximal tubular cell apoptosis.^10, 11, 12^ Flow cytometry of DNA content showed that in our system, albumin increased the number of hypodiploid apoptotic proximal tubular cells in a dose- and time-dependent manner (Figures 4a–c). An increased rate of apoptosis was already evident at 24 h and further increased at 48 h of exposure to albumin (Figures 4a–c). Staining with 7-AAD/annexin V staining confirmed the increase in cell death (Figure 4d).

### Albumin induces a time-dependent increase in BASP1 mRNA and protein expression in tubular cells

**Figure 5:**
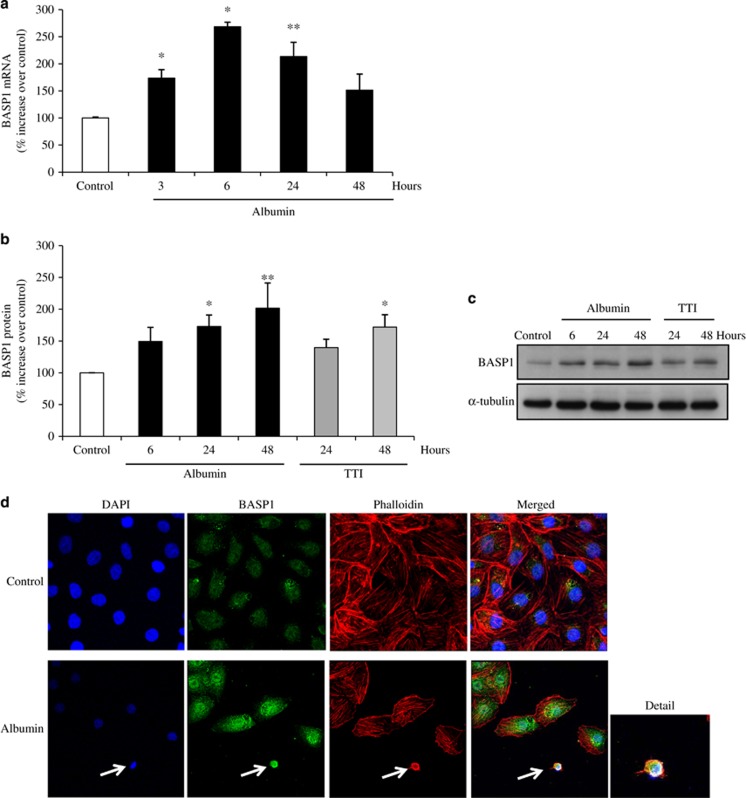
Albumin increases BASP1 expression in human proximal tubular epithelial cells. (**a**) Real-time RT-PCR analyses of BASP1 mRNA expression in cells cultured in the presence of albumin (100 mg/ml). Mean±S.D. of four independent experiments. *P<0.005 *versus* control, **P<0.01 *versus* control. (**b**) Quantification of western blot results. Cells were exposed to 100 mg/ml albumin. Mean ±S.D. of four independent experiments. *P<0.03 *versus* control, **P<0.005 *versus* control. (**c**) Representative western blot of BASP1. BASP1 expression was also increased by an apoptosis-inducing cytokine cocktail (100 ng/ml TWEAK, 30 ng/ml TNF*α* and INF-*γ* 30 U/ml). (**d**) Confocal microscopy confirmed increased BASP1 (green) expression in cells exposed to albumin (100 mg/ml for 24 h). Colocalization of BASP1 was further explored in cells undergoing albumin-induced apoptosis. BASP1 localized to the cell periphery in apoptotic cells with pyknotic nuclei (DAPI, blue), where it colocalized with phalloidin-stained actin (red) rings (arrow and detail)

We examined the influence of albumin on BASP1 expression in tubular cells. At concentrations that promoted tubular cell apoptosis, albumin increased BASP1 mRNA (Figure 5a) and protein (Figures 5b and c) expression in a time-dependent manner. BASP1 expression was also increased by the lethal combination of proinflammatory cytokines TWEAK/TNF/interferon-*γ* (INF*-γ*).^23^ Confocal microscopy localized the increased BASP1 expression in albumin-treated cells mainly to the perinuclear area. A peripheral location near the cell membrane was more conspicuous in albumin-treated apoptotic cells, where it colocalized with actin (Figure 5d). This is consistent with the peripheral localization of BASP1 in high-glucose-exposed apoptotic tubular cells.^19^ The observation that albumin increased BASP1 expression in tubular cells, suggests that the increased tubular BASP1 expression observed *in vivo* in proteinuric diseases may be a direct manifestation of albumin cytotoxicity against tubular cells.

### Inhibition of BASP1 expression protects against albumin-induced apoptosis

**Figure 6:**
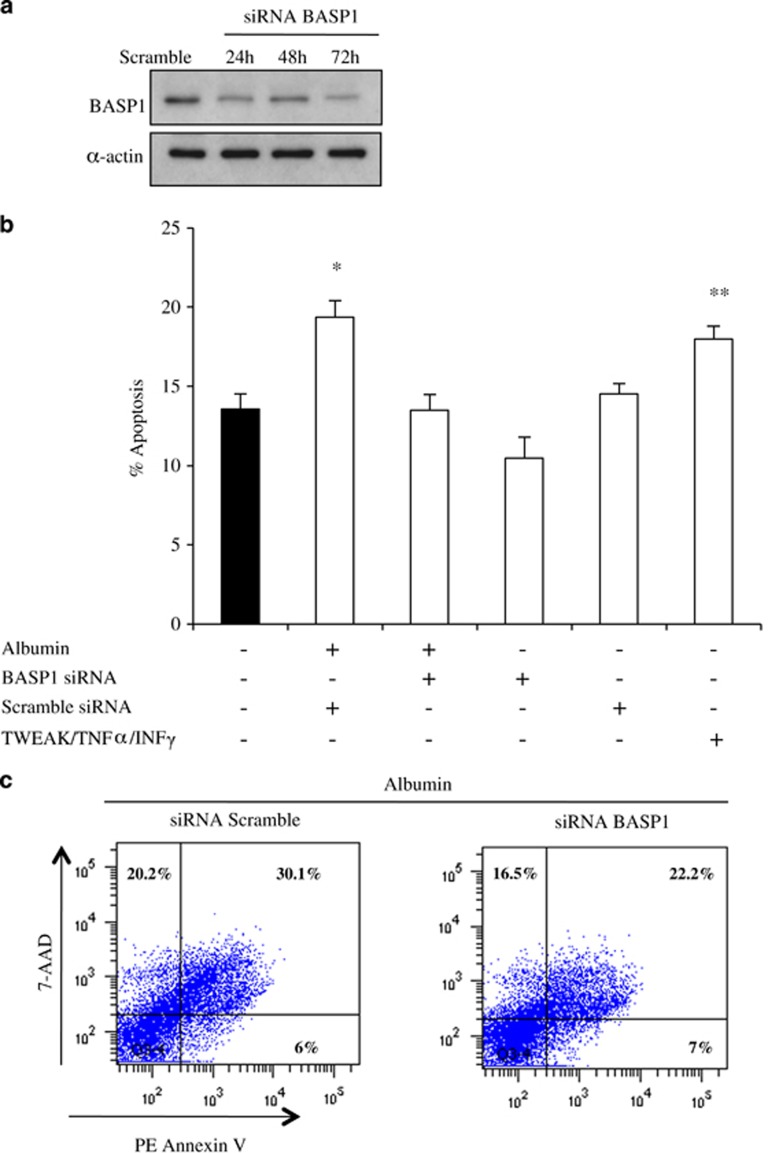

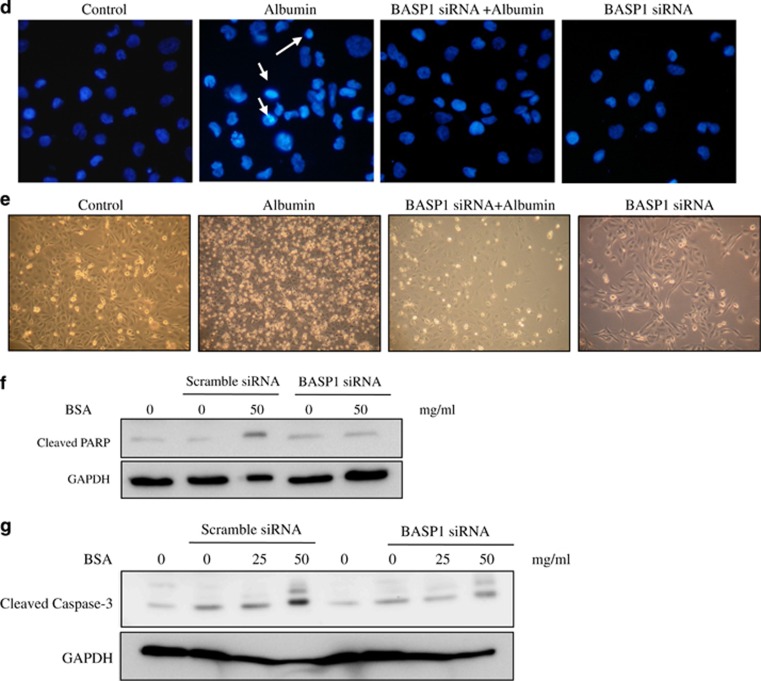
Inhibition of BASP1 expression prevents apoptosis in human tubular epithelial cells. (**a**) BASP1 siRNA decreases the expression of BASP1 protein. Representative experiment. BASP1 mRNA expression was decreased by 70% (not shown). (**b**) Cells transfected with BASP1 siRNA were protected from albumin-induced apoptosis. Cell death was assessed by flow cytometry of DNA content (hypodiploid cells suggestive of apoptosis) after culture in the presence of albumin (100 mg/ml) for 48 h. Mean±S.D. of three independent experiments. *P<0.015 *versus* control or *versus* albumin+BASP1 siRNA. **P<0.02 *versus* control. (**c**) BASP1 knockdown with BASP1 siRNA protects from albumin-induced cell death (100 mg/ml) as assessed by flow cytometry following staining with annexin V and 7-AAD for 48 h. Representative experiment. (**d**) Morphological evidence of apoptosis (arrows: fragmented, shrunk, bright nuclei) in permeabilized cells stained with DAPI (blue) (original magnification × 200). Cells were treated for 48 h with vehicle or 100 mg/ml albumin in the presence or absence of BASP1 siRNA. (**e**) Phase contrast microscopic photographs (original magnification × 200). Note numerous detached cells among those exposed to 100 mg/ml albumin for 48 h in the absence of BASP1 siRNA. (**f**) BASP1 targeting prevents PARP processing in tubular cells exposed to albumin. Representative western blot. (**g**) BASP1 targeting prevents caspase-3 activation in tubular cells exposed to albumin. Representative western blot

To address the role of BASP1 in albumin-induced apoptosis, we used a siRNA approach. Western blot and immunofluorescence confirmed efficient gene silencing by BASP1-specific siRNA (Figure 6a,Supplementary Figure 1). BASP1 targeting protected from albumin-induced apoptosis quantified as percentage of hypodiploid cells by flow cytometry of cell DNA content (Figure 6b). Decreased cell death was also observed by 7-AAD/annexin V staining (Figure 6c). Morphological studies disclosed the presence of pyknotic nuclei characteristic of apoptosis among cells exposed to albumin and protection of albumin-exposed cells from apoptosis by BASP1 siRNA (Figure 6d). By phase contrast microscopy, many detached, small-sized floating cells were observed in cells exposed to albumin (Figure 6e). This is consistent with the cell detachment and fragmentation characteristic of apoptosis.^17^ Surviving cells exposed to albumin and treated with BASP1 siRNA looked healthy by contrast-phase microscopy (Figure 6e). Albumin also increased Poly(ADP-ribose) polymerase (PARP) processing and caspase-3 activation and this was decreased by BASP1 siRNA targeting (Figures 6f and g and Supplementary figure 2). These results suggest that BASP1 targeting protects from albumin-induced apoptosis.

### BASP1 overexpression induces apoptosis in tubular epithelial cells

**Figure 7:**
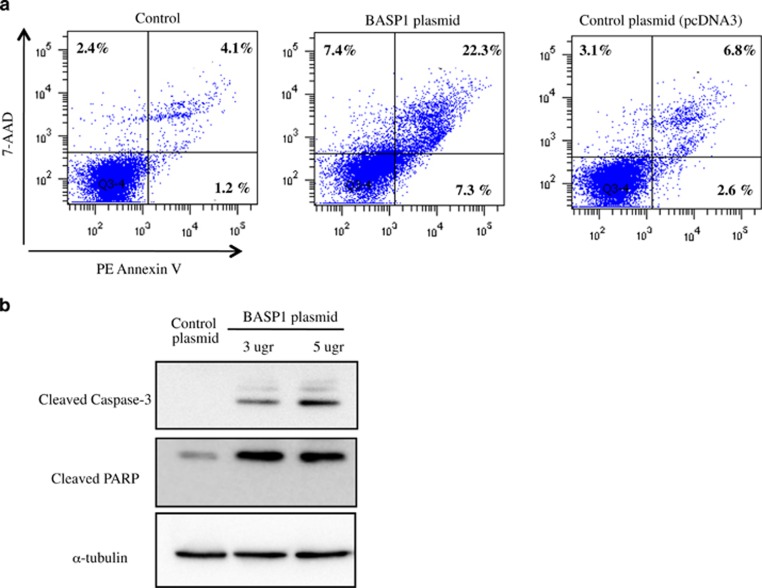
BASP1 overexpression promotes human tubular epithelial cell apoptosis. (**a**) Tubular cells were transfected with BASP1 (5 *μ*g) or control vector (5 *μ*g pcDNA3) for 24 h and cell death was assessed by flow cytometry following staining with annexin V and 7-AAD (representative experiment). (**b**) BASP1 overexpression induces PARP processing and caspase-3 activation at 24 h. Representative western blot

We had previously observed that BASP1 overexpression promotes apoptosis in tubular epithelial cells.^19^ We have expanded this observation by assessing BASP1-induced apoptosis by techniques not used in the previous publication. Thus, overexpression of BASP1 increased the percentage of apoptotic cells as assessed by flow cytometry (Figure 7a). BASP1 overexpressing cells displayed PARP processing and caspase-3 activation (Figure 7b).

## Discussion

The main finding of this study is that BASP1 is an intracellular mediator of albumin-induced tubular cell death. Tubular cell BASP1 is upregulated in experimental and human proteinuric kidney disease, supporting the clinical relevance of the observation. Thus, we have provided functional evidence supporting the concept that BASP1 is a novel therapeutic target to protect tubular cells from the adverse effects of excess albumin. This observation may lead to the development of new therapeutic approaches to prevent CKD progression in patients with persistent residual albuminuria despite the prescription of current anti-proteinuric drugs.

BASP1 is a multifunctional protein that may behave as a transcription factor, may have lipid raft and cytoskeletal organizational properties, and may induce single channel cation selective currents across negatively charged planar lipid bilayers.^24, 25, 26, 27^ In addition, BASP1 was recently characterized as a death promoter in tubular cells exposed to high glucose concentrations, but not in apoptosis induced by a combination of proinflammatory cytokines.^19^

BASP1 is expressed in kidney cells.^25, 19^ Podocytes are the main site of BASP1 expression in normal kidneys.^25^ In podocytes, BASP1 localizes to nuclei and regulates the transcription factor activity of WT-1. By contrast, normal tubular cells express very low or undetectable amounts of cytoplasmic BASP1. These differences in protein expression and protein location are consistent with potentially diverse roles of BASP1 in different cell types.

BASP1 is a member of the GAP-43, MARCKS and BASP1/CAP-23 (GMC) family of functionally related proteins that share an effector domain, acylation-mediated targeting to the cell membrane and association with the cortical cytoskeleton.^26^ A role in apoptosis has been suggested for GAP-43 and *Xenopus* MARCKS-related protein in non-renal cells.^28, 29, 30^ Enforced BASP1 overexpression induces apoptosis in tubular epithelium.^19^ Furthermore, endogenous BASP1 expression is increased by either a lack of survival factors or by the presence of certain lethal stimuli such as high glucose or a cytokine combination (TWEAK/TNF*α*/INF-*γ*).^19^ Albumin is a novel, pathophysiologically relevant stimulus that promotes tubular cell apoptosis, BASP1 upregulation and BASP1 redistribution to the cell cortex in apoptotic cells, where BASP1 colocalizes with the apoptotic actin ring of apoptotic cells. In addition to being pathophysiologically relevant, albuminuria is the current standard to assess CKD progression risk in the clinic.^2^ Thus, albuminuria is quantified in CKD patients during routine clinical care. This allows the easy identification in daily clinical practice of patients that might benefit from nephroprotective strategies that protect tubular cells from the adverse effects of albumin. In this regard, siRNA targeting of BASP1 protected tubular cells from albumin-induced apoptosis. The present report does not explore the molecular mechanisms of BASP1 promotion of apoptosis, because in tubular cells, BASP1-induced apoptosis was already shown to be prevented by Bax antagonists and caspase inhibitors, suggesting that BASP1 has a role upstream of mitochondrial injury and caspase activation. By contrast to the requirement for BASP1 for tubular cell apoptosis induced by albumin, cytokine (TWEAK/TNF*α*/INF-*γ*)-induced apoptosis did not require BASP1.^19^ Interestingly, cytokine-induced tubular cell apoptosis was recently shown to have also features of necroptosis.^31^

Increased tubular BASP1 expression was observed in non-diabetic human and experimental albuminuric nephropathies. This suggests that the increased tubular BASP1 previously observed in diabetic nephropathy, which is also an albuminuric nephropathy, cannot be solely attributed to high glucose levels.^19^ Thus, increased tubular BASP1 is also observed when albuminuria is present and glucose levels are normal. In this regard, according to the Kidney & Urinary Pathway Knowledge Base (KUPKB, www.kupkb.org; accessed 25 February 2014), BASP1 protein is increased in urine from patients with type 2 diabetes with microalbuminuria as compared with those with normoalbuminuria,^32^ despite both groups displaying high glucose levels. These findings are consistent with a role of albumin as an inducer of tubular cell BASP1 expression *in vivo*. Moreover, data collected in the Nephromine database showed that in 21 patients with membranous nephropathy (eGFR 85±40 ml/min/1.73 m^2^, age 54±18 years, 12 males/9 females), tubulointerstitial BASP1 mRNA expression was found to be 1.204-fold (*P*=0.012) that of healthy living kidney donors. For comparison, non-proteinuric conditions such as thin basement membrane disease (fold-change 1.068) and tumor nephrectomy (fold-change −1.042) did not significantly differ from healthy living kidney donors. (http://www.nephromine.org/; accessed 2 June 2014).^33^

In rat nephrotic syndrome, BASP1 protein expression increased to a higher degree that mRNA expression. Discrepancies between mRNA and protein expression are not unusual. There are several potential explanations for this observation. The peak mRNA expression may have occurred at an earlier time point. Alternatively, BASP1 protein half-life may be increased. In this regard, posttranslational modifications of BASP1 may underlie differences in location, function and, possibly, half-life. BASP1 may undergo a series of posttranslational modifications such as myristoylation, sumoylation, caspase-dependent processing or other proteolytic processes.^34, 35, 36, 37^ The transcription factor function of BASP1 is regulated by sumoylation^34^ and myristoylation.^35^ An N-terminally myristoylated form of BASP1 regulates actin cytoskeleton dynamics in neurons. In neurons, BASP1 is localized mainly in the lipid rafts of synaptic vesicles and plasma membranes, where it could participate in the transport and anchoring of glutamic acid decarboxylase isoforms (GAD) GAD65 and GAD67.^20^ More in line with the current report, recent data have linked BASP1 to tumor suppression.^38, 39, 40^ The proapoptotic role of BASP1 might contribute to tumor suppression, although this possibility has not been formally explored. Increased BASP1 expression has also been observed in other inflammatory conditions, although the role of BASP1 in these conditions was not explored. Thus, gene expression profiling studies disclosed increased BASP1 expression in human dermatitis herpetiformis^41^ and in goat mammary glands infected with *S. aureus*.^42^ A better grasp of the determinants of BASP1 intracellular localization and function may contribute to the design of BASP1-targeting therapies. The unique localization and set of transporters of proximal tubular cells may be advantageous to design therapies specifically targeting these cells.^43, 44, 45^ Thus, we may take advantage of the albumin overload of proximal tubular cells in proteinuric states and their capacity to recover molecules from the glomerular ultrafiltrate, as well as of the high blood flow of the kidneys (one-fourth of the heart output by volume) and the magnitude of glomerular filtration (over 100 ml/min) to target anti-BASP1 molecules specifically to tubular cells.

The data presented in this study clearly show that exposure to albumin, as may occur *in vivo* in proteinuric nephropathies, increases BASP1 expression and promotes BASP1-dependent tubular cell apoptosis. This information may be used to design novel therapeutic approaches that slow CKD progression by protecting tubular cells from the adverse consequences of albuminuria in patients not fully responding to current anti-proteinuric agents. These novel approaches may include interfering with BASP1 function in tubular epithelium by using specific siRNAs.

## Materials and Methods

### Cell culture and reagents

HK-2 human proximal tubular epithelial cells (ATCC, Rockville, MD, USA) were grown on RPMI 1640 (Life Technologies, Grand Island, NY, USA) with 10% heat-inactivated FBS, 2 mM glutamine, 100 U/ml penicillin, 100 *μ*g/ml streptomycin, 5 *μ*g/ml insulin, 5 *μ*g/ml transferrin, 5 ng/ml sodium selenite and 5 ng/ml hydrocortisone in 5% carbon dioxide at 37 °C. For experiments, cells were cultured in serum-free media 24 h prior to addition of stimuli and throughout the experiment. Exposure of cultured tubular cells to bovine serum albumin (Sigma, St. Louis, MO, USA) was used as a surrogate for the *in vivo* exposure of tubular cells to albumin in proteinuric nephropathies. Recombinant human soluble TWEAK (Millipore, Billerica, MA, USA) was used at 100 ng/ml, murine TNF-*α* (PrePotech, London, UK) at 30 ng/ml and INF-*γ* (PrePotech, London, UK) at 30 U/ml.

### Cell death and apoptosis

Cells were cultured to subconfluence in 12-well plates. Apoptosis was assessed by flow cytometry of DNA content and nuclear morphology. For assessment of apoptosis, adherent cells were pooled with spontaneously detached cells, and stained in 100 *μ*g/ml propidium iodide, 0.05% NP-40, 10 *μ*g/ml RNAse A in PBS at 4 °C for >1 h. This assay permeabilizes the cells. Permeabilization allows entry of propidium iodide into all cells, dead or alive. Apoptotic cells are characterized by a lower DNA content (hypodiploid cells) because of nuclear fragmentation. Thus, this assay is not based on the known ability of propidium iodide to enter dead cells. The percentage of apoptotic cells with decreased DNA content (A_o_) was counted by flow cytometry using BD CellQuest Software (BD Biosciences, San Diego, CA, USA).^46^ As a positive control, apoptosis was induced by exposure to a lethal cytokine cocktail (30 ng/ml TNF*α*, 100 ng/ml TWEAK and 30 UI/ml INF-*γ*) for 48 h. Nuclei of formalin-fixed cells were stained with DAPI (Sigma) to observe the typical morphological changes, as previously described.^19, 46^

The PE Annexin V Apoptosis Detection Kit I (BD Pharmingen, San Diego, CA, USA) was used to assess both apoptosis and necrosis by flow cytometry. This method discriminates between early apoptotic (7-AAD-negative, annexinV-positive), and late apoptotic or necrotic cells (7-AAD-positive, annexinV-positive).

### Western blot analysis

Tissue and cell samples were homogenized in lysis buffer,^47^ then separated by 10 or 12% SDS-PAGE under reducing conditions and transferred to PVDF membranes (Millipore, Bedford, MA, USA), blocked with 5% skimmed milk in PBS/0.5% v/v Tween 20 for 1 h, and washed with PBS/Tween. Primary antibody was goat polyclonal anti-BASP1 (1 : 500, Santa Cruz, Santa Cruz, CA, USA), rabbit polyclonal anti-cleaved caspase-3 (1 : 1000; Cell Signaling, Hertfordshire, UK), rabbit monoclonal anti-cleaved PARP (1 : 1000, Abcam, Cambridge, UK), mouse anti-tubulin monoclonal antibody (1 : 5000, Sigma) or mouse GAPDH (Millipore, Billerica) followed by incubation with horseradish peroxidase-conjugated secondary antibody (1 : 2000, Amersham, Aylesbury, UK). Blots were developed with the enhanced chemiluminescence method following the manufacturer's instructions (Amersham). Levels of expression were corrected for minor differences in loading.

### Quantitative reverse transcription-polymerase chain reaction

One microgram RNA isolated by Trizol (Invitrogen, Paisley, UK) was reverse-transcribed with High Capacity cDNA Archive Kit and real-time PCR was performed on a ABI Prism 7500 PCR system (Applied Biosystems, Foster City, CA, USA) using the DeltaDelta Ct method.^46^ Expression levels are given as ratios to GAPDH. Pre-developed primer and probe assays were from Applied Biosystems.

### Transient transfection

For transient transfection, cells were plated at a density of 8 × 10^4^ cells/plate in six-well plates (Costar, Cambridge, MA, USA) with RPMI 1640 (10% FBS) 24 h before transfection. Cells were transfected with 5 *μ*g of BASP1 containing plasmid or empty vector (pcDNA3) using FuGENE 6 (Roche, Indianapolis, IN, USA) according to the manufacturer's protocol. Real-time RT-PCR analyses of BASP1 mRNA in cells transfected with BASP1 confirmed a 300-fold BASP1 overexpression.^19^

### Transfection of small-interfering RNA

Cells were grown in six-well plates (Costar) and transfected with a mixture of 20 nmol/ml BASP1 siRNA (Ambion, Applied Biosystems), Opti-MEM I Reduced Serum Medium and siPORT *Amine* Transfection Agent (Ambion, Applied Biosystems).^19^ After 18 h, cells were washed and cultured for 48 h in complete medium and serum-depleted for 24–48 h before stimulation. These time points were selected from a time-course of BASP1 protein expression in response to siRNA. A negative control scrambled siRNA provided by the manufacturer did not reduce BASP1 protein.

### Confocal microscopy

Cells plated onto Labtek slides (Nunc, Naperville, IL, USA) were fixed in 4% paraformaldehyde and permeabilized in 0.2% Triton X-100 in PBS for 10 min each. After washing in PBS, cells were incubated overnight at 4 °C with rabbit polyclonal anti-BASP1 antibody (1 : 100, Abcam), followed by incubation with anti-rabbit Alexa Fluor 488 (1 : 300, Invitrogen). After washing, cells were mounted in 70% glycerol in PBS, and analyzed with a DM-IRB confocal microscope (Leica DM, Bannockburn, IL, USA).^46^ F-actin was stained with 800 *μ*M TRITC-phalloidin (Sigma) in the dark for 30 min at room temperature. Apoptosis was characterized by morphologic and functional criteria. Nuclei cells were stained with DAPI (Vector Laboratories, Inc., Burlingame, CA, USA) to observe the typical morphological changes.

### Animal model

Two groups of 10-week-old Wistar Kyoto rats (Criffa, Barcelona, Spain) were studied (*n*=9/group). Nephrotic syndrome was induced by a single i.v. injection of 150 mg/kg PAN (Sigma).^48^ Control rats received vehicle (Saline). Rats were killed at day 2 and day 10 after PAN injection following a 24 h urine collection in metabolic cages. Kidneys were perfused *in situ* with cold saline before removal. One kidney from each rat was fixed in buffered formalin, embedded in paraffin and used for immunohistochemistry. The other kidney was snap-frozen in liquid nitrogen for RNA and protein studies. Albuminuria was determined by ELISA, using rat albumin as a standard (Celltrend, Luckenwalde, Germany). All studies were performed in accordance with the European Union normative.

### Immunohistochemistry and immunofluorescence

Kidney samples were obtained from the IIS-FJD Biobank (Madrid, Spain). Samples were obtained by percutaneous renal biopsy from patients undergoing diagnostic evaluation for proteinuric nephropathy at the Division of Nephrology (IIS-FJD) that donated for research the remnant tissue following diagnostic evaluation. Kidney biopsy samples from six patients with a diagnosis of membranous nephropathy were studied. Age (mean±S.D.) 61±4, sex 3 male/3 female, creatinine 2.1±0.9 mg/dl, albuminuria 4897±330 mg/g creatinine, HbA1C level<6.0%. Control human kidney specimens were taken from normal portions of renal tissue from patients who underwent surgery because of localized renal tumors. Immunohistochemistry was carried out in paraffin-embedded tissue sections of 5 *μ*m thickness as previously described.^19^ Primary antibody was rabbit polyclonal anti-BASP1 (1 : 50, Abcam). Sections were counterstained with Carazzi's hematoxylin. Negative controls included incubation with a non-specific immunoglobulin of the same isotype as the primary antibody. The local Ethics Committee approved the study protocol and informed consent was obtained. Rat tissue immunohistochemistry was performed as described for human tissue.

To correlate apoptosis with BASP1 expression in rat kidney, a double-labeling immunoflurescence was used. Apoptosis was assayed by deoxynucleotidyl-transferase-mediated dUTP nick-end labeling (*In Situ* Cell Death Detection Kit; Roche) according to the manufacturer's instructions. Next, anti-BASP1 (1 : 100, Abcam) was added followed by Alexa-488-conjugated secondary antibody as previously described.^47^

### Statistics

Data are expressed as mean±standard error of media. Mann-Whitney, two-sided *t*-test or one-way ANOVA were applied to assess differences between groups. A *P*-value <0.05 was considered statistically significant.

## Abbreviations

7-AAD: 7-aminoactinomycin D
BASP1: brain abundant signal protein 1
CAP-23: cortical cytoskeleton-associated protein 23
CKD: chronic kidney disease
DAPI: 4′,6-diamidino-2-phenylindole
DNA: Deoxyribonucleic acid
eGFR: estimated Glomerular Filtration Rate
FBS: Fetal bovine serum
GAD: glutamic acid decarboxylase
GAP-43: Growth Associated Protein 43
GAPDH: glyceraldehyde-3-phosphate dehydrogenase
HK2: human kidney 2
IFN*γ*: Interferon gamma
IIS-FJD: Instituto de Investigación Sanitaria, Fundación Jiménez Díaz
KUPKB: kidney & Urinary Pathway Knowledge Base
MARCKS: Myristoylated alanine-rich C-kinase substrate
mRNA: Messenger Ribonucleic acid
PAN: Puromycin aminonucleoside
PARP: Poly(ADP-ribose) polymerase
PKC: Protein kinase C
PVDF: polyvinylidene difluoride
RPMI: Roswell Park Memorial Institute
SDS-PAGE: sodium dodecyl sulfate polyacrylamide gel electrophoresis
siRNA: Small interfering Ribonucleic acid
TNF: Tumor necrosis factor
TUNEL: deoxynucleotidyl-transferase-mediated dUTP nick-end labeling
TWEAK: Tumor necrosis factor-like weak inducer of apoptosis
WT-1: Wilms tumor 1
