# Supplementary figures and images for: Albumin-induced apoptosis of tubular cells is modulated by BASP1
Source: Cell Death Dis. 2015 Feb 12;6(2):e1644–. doi: 10.1038/cddis.2015.1 (PMC4669784; doi:10.1038/cddis.2015.1)

**Supplementary Figure 1**

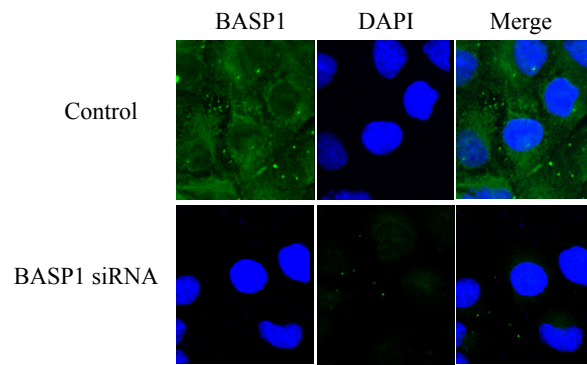

Supplementary Figure 2

A)

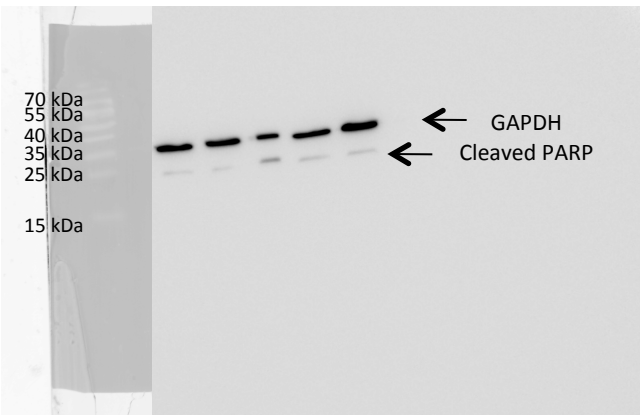

B)

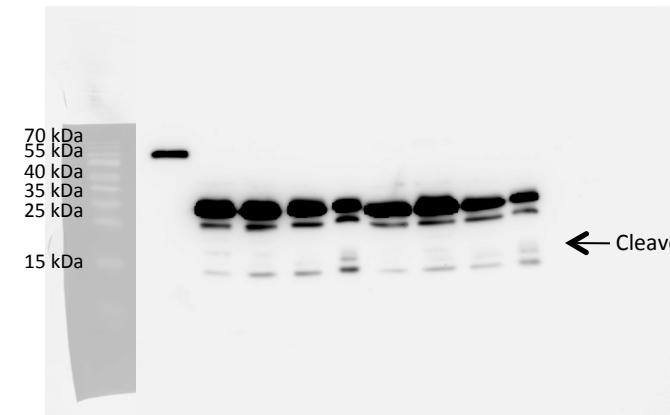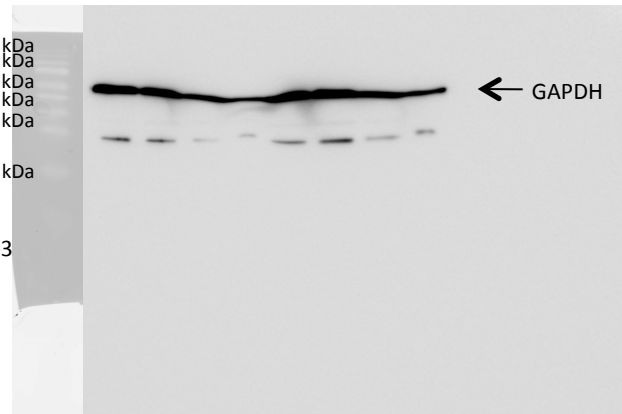

C)

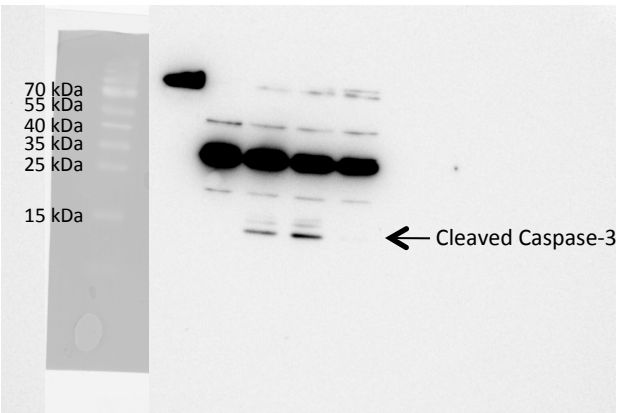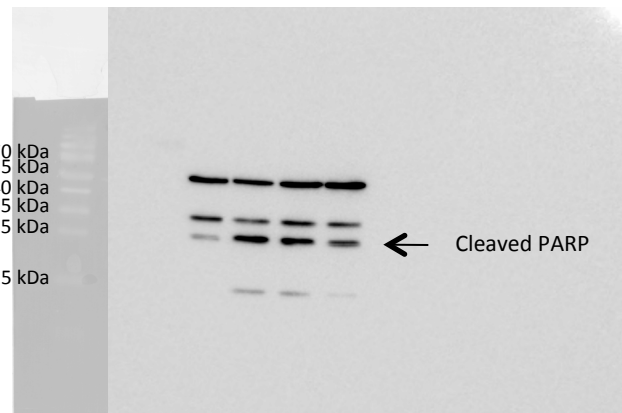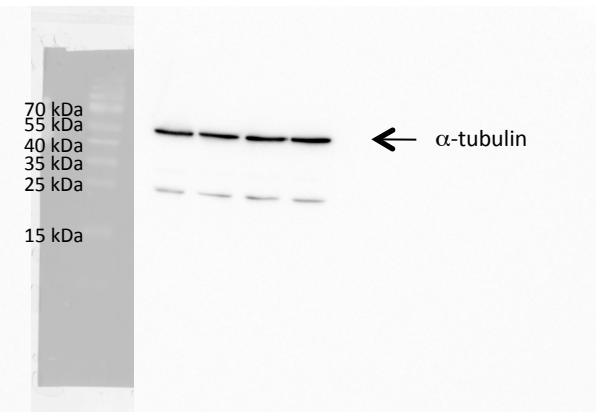

Supplement: Supplementary Figures [file cddis20151x1.pdf]
