# Supplementary material for: Albumin-induced apoptosis of tubular cells is modulated by BASP1
Source: Cell Death Dis. 2015 Feb 12;6(2):e1644–. doi: 10.1038/cddis.2015.1 (PMC4669784; doi:10.1038/cddis.2015.1)
Supplement: Supplementary Figure Legends [file cddis20151x2.doc]

**SUPPLEMENTARY** **FIGURE LEGENDS**

**Supplementary** **figure 1. BASP1 immunofluorescence.** BASP1 siRNA decreases the expression of BASP1 protein. Cells were transfected with BASP1 siRNA or Scramble siRNA and stained with rabbit polyclonal anti-BASP1 antibody (1:00, Abcam), followed by incubation with anti-rabbit Alexa Fluor 488 (1:300, Invitrogen) antibody and counterstained with DAPI. Note BASP1 staining in scrambled siRNA transfected cells but not in BASP1 siRNA transfected cells.

**Supplementary** **figure 2. Whole western blots corresponding to figures 6F (A), 6G (B) and 7B (C).**
